# Association between aging-dependent gut microbiome dysbiosis and dry eye severity in C57BL/6 male mouse model: a pilot study

**DOI:** 10.1186/s12866-021-02173-7

**Published:** 2021-04-09

**Authors:** Chang Ho Yoon, Jin Suk Ryu, Jayoon Moon, Mee Kum Kim

**Affiliations:** 1 Department of Ophthalmology, Seoul National University College of Medicine, 103 Daehak-ro, Jongno-gu, Seoul, 03080 Republic of Korea; 2 Department of Ophthalmology, Seoul National University Hospital, 101 Daehak-ro, Jongno-gu, Seoul, 03080 Republic of Korea; 3 Laboratory of Ocular Regenerative Medicine and Immunology, Biomedical Research Institute, Seoul National University Hospital, 101 Daehak-ro, Jongno-gu, Seoul, 03080 Republic of Korea

**Keywords:** Dry eye, Aging, Microbiome, *Paraprevotella*, 16S rRNA

## Abstract

**Background:**

While aging is a potent risk factor of dry eye disease, age-related gut dysbiosis is associated with inflammation and chronic geriatric diseases. Emerging evidence have demonstrated that gut dysbiosis contributes to the pathophysiology or exacerbation of ocular diseases including dry eye disease. However, the relationship between aging-related changes in gut microbiota and dry eye disease has not been elucidated. In this pilot study, we investigated the association between aging-dependent microbiome changes and dry eye severity in C57BL/6 male mice.

**Results:**

Eight-week-old (8 W, *n* = 15), one-year-old (1Y, *n* = 10), and two-year-old (2Y, *n* = 8) C57BL/6 male mice were used. Dry eye severity was assessed by corneal staining scores and tear secretion. Bacterial genomic 16 s rRNA from feces was analyzed. Main outcomes were microbiome compositional differences among the groups and their correlation to dry eye severity. In aged mice (1Y and 2Y), corneal staining increased and tear secretion decreased with statistical significance. Gut microbiome α-diversity was not different among the groups. However, β-diversity was significantly different among the groups. In univariate analysis, phylum *Firmicutes*, *Proteobacteria,* and *Cyanobacteria*, *Firmicutes*/*Bacteroidetes* ratio, and genus *Alistipes*, *Bacteroides*, *Prevotella*, *Paraprevotella*, and *Helicobacter* were significantly related to dry eye severity. After adjustment of age, multivariate analysis revealed phylum *Proteobacteria*, *Firmicutes*/*Bacteroidetes* ratio, and genus *Lactobacillus*, *Alistipes*, *Prevotella*, *Paraprevotella*, and *Helicobacter* to be significantly associated with dry eye severity.

**Conclusions:**

Our pilot study suggests that aging-dependent changes in microbiome composition are related to severity of dry eye signs in C57BL/6 male mice.

**Supplementary Information:**

The online version contains supplementary material available at 10.1186/s12866-021-02173-7.

## Background

Dry eye disease is a multifactorial disorder in which the tear film homeostasis is lost, accompanied by several ocular symptoms. Tear film instability and ocular surface inflammation may contribute to the disease [1, 2]. Given that the prevalence of dry eye disease increases after middle age, aging is one of the critical risk factors for dry eye disease [3, 4]. As the elderly population increases, socio-economic costs are expected to proportionally increase in the near future [5]. Ocular surface is an exposed mucosa constantly subject to external stimuli. Tear film instability induces hyperosmolar stress in ocular surface epithelium, which subsequently acts as an initial precipitating factor for dry eye disease. Hyperosmolar stress promotes the expression of inflammatory cytokines in the damaged ocular surface epithelium [1]. The inflammatory cytokines may recruit innate immune cells, activate dendritic cells and stimulate T helper 1 (Th1) and 17 (Th17) cells that infiltrate the ocular surface and lacrimal glands [1, 6]. Regulatory T cell (T_reg_) dysfunction is also involved in the progression to chronic dry eye disease [7].

Gut microbes influence the maturation and development of the immune system, interact with the nervous system, and maintain metabolic homeostasis [8–10]. Emerging evidence have implied that gut dysbiosis contributes to the pathophysiology or exacerbation of intestinal diseases as well as systemic diseases including systemic lupus erythematosus and rheumatoid arthritis [11–14]. Moreover, recent studies have demonstrated that alterations in gut microbiota may lead to several ocular diseases including autoimmune uveitis, age related macular degeneration, and dry eye associated with Sjögren’s syndrome [15–18]. A recent review also indicates that gut microbiota may interact with the eye [19].

The gut microbiota changes with aging and this aging-related microbiota alteration is associated with inflammaging and chronic geriatric diseases [20]. Given that the gut dysbiosis is associated with dry eye disease [19], aging-related microbiome dysbiosis may also contribute to aging-related dry eye disease. Therefore, in this pilot study, we aimed to investigate (1) whether aging-related dysbiosis is present in C57BL/6 (B6) male mice and (2) which taxa that altered with aging may be associated with the severity of dry eye. These results will open the door for microbiota modulation in aging-related dry eye disease as one of the treatment options.

## Results

**Fig. 1:**
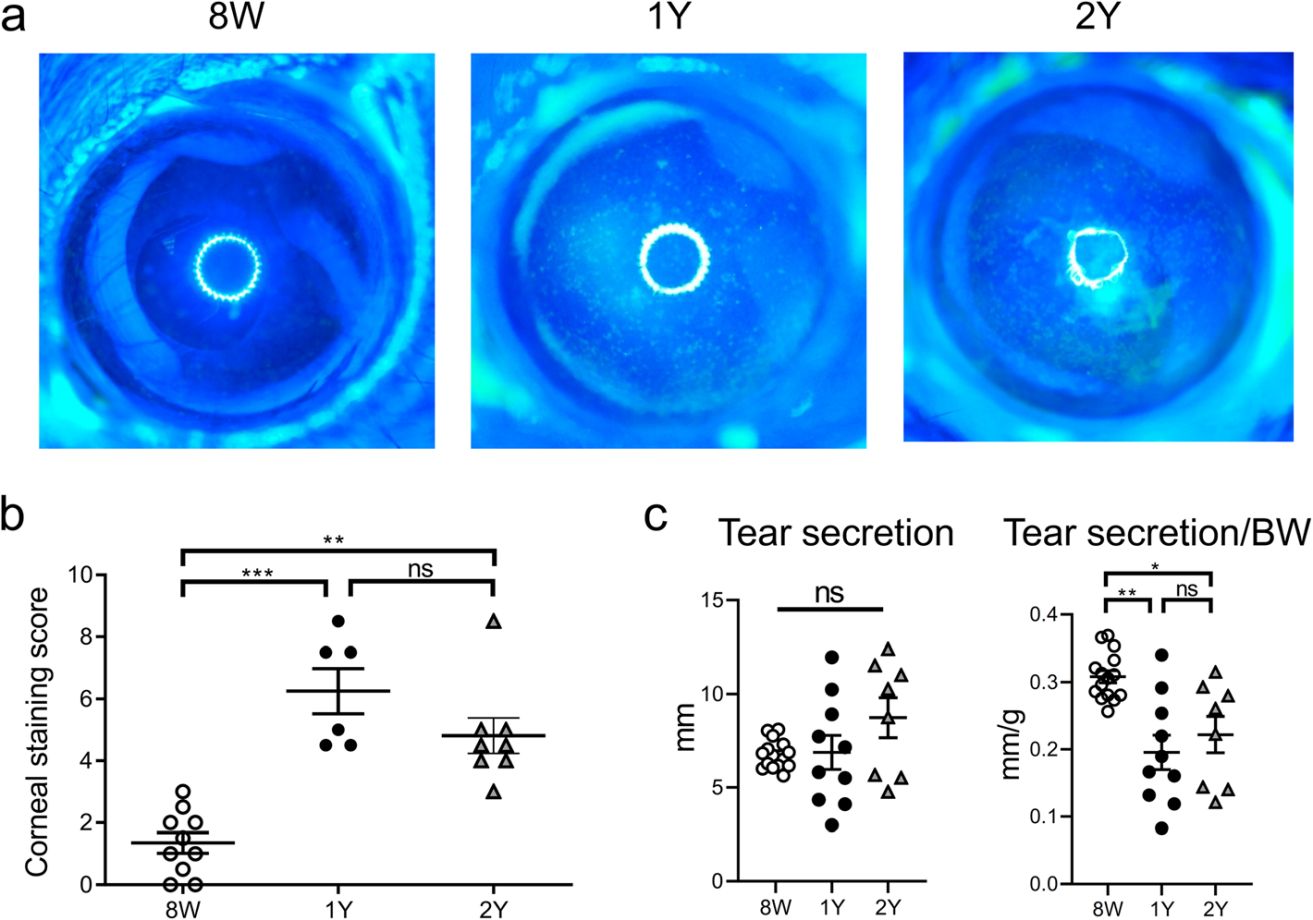
Corneal staining and tear secretion of the 8 W, 1Y, and 2Y groups. **a** Representative images of corneal fluorescein staining. **b** The National Eye Institute corneal staining score was significantly higher in one-year-old (1Y) and two-year-old (2Y) mice than in eight-week-old (8 W) mice (all *p* < 0.01; Kruskal–Wallis test followed by Dunn’s post hoc test; *n* = 10, 6, and 8 for 8 W, 1Y, and 2Y mice, respectively). **c** Tear secretion did not change over time (all *p* > 0.05; Kruskal–Wallis test). However, when the level was normalized by body weight (BW), the normalized value was lower in both 1Y and 2Y groups than in the 8 W group (all *p* < 0.05; Kruskal–Wallis test followed by Dunn’s post hoc test; *n* = 15, 10, and 8 for 8 W, 1Y, and 2Y mice, respectively). ns, not significant; *p < 0.05, **p < 0.01, and ***p < 0.001. Data are presented as means ± standard error

First, we discovered that dry eye developed according to age in B6 male mice. The mean (± standard deviation (SD)) corneal staining scores (punctate epithelial erosions) of eight-week-old (8 W; young), one-year-old (1Y; middle aged), and two-year-old (2Y; elderly) groups were 1.35 ± 1.03, 6.25 ± 1.78, and 4.81 ± 1.62, respectively (Fig. 1a-b). The scores of the 1Y and 2Y groups were significantly higher than that of the 8 W group (*p* < 0.001 and *p* = 0.005, respectively); however, there was no difference between the 1Y and 2Y groups (*p* > 0.999; Kruskal–Wallis test followed by Dunn’s post hoc test) (Fig. 1a-b). The amount of tear secretion was not different among the groups; whereas, the correction value according to body weight (BW), which is also clinically relevant, was significantly lower in both 1Y and 2Y groups than in the 8 W group (Fig. 1c; *p* = 0.003 and *p* = 0.044, respectively; Kruskal–Wallis test followed by Dunn’s post hoc test). Supplementary Table 1 shows all measurements of each mouse.

**Fig. 2:**
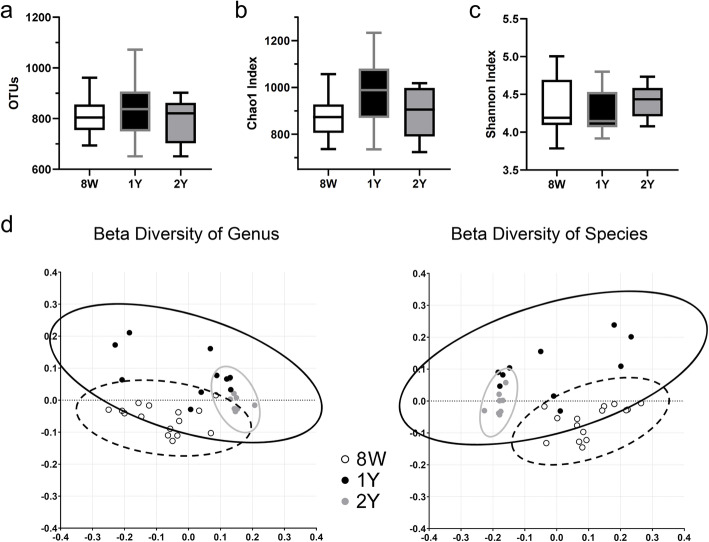
Alpha and beta diversity analysis of the 8 W, 1Y, and 2Y groups. **a** Observed operational taxonomic unit (OTU) count, (**b**) Chao 1 index, (**c**) Shannon diversity index. There were no significant differences among the groups in OTU, Chao1 and Shannon index (all *p* > 0.05; Kruskal–Wallis test). **d** β-diversity of genus and species analyzed by UniFrac principal coordinates analysis revealed to have significant distances between each group (*p* = 0.001, 8 W vs. 1Y; *p* = 0.001, 8 W vs. 2Y; and *p* = 0.009, 1Y vs. 2Y; Permutational multivariate analysis of variance (PERMANOVA)). Bars indicate maximum and minimum values

Next, we compared each groups’ gut microbiome. Mean (± SD) reads per sample was 44,711 (± 10,442) and individual samples ranged from 28,903 to 79,815 reads. The number of operational taxonomic units (OTUs) of each group were not different (*p* = 0.777; Kruskal–Wallis test; Fig. 2a). Chao 1 index and Shannon index did not show any significant differences among the groups (*p* = 0.207 and *p* = 0.395, respectively; Kruskal–Wallis test; Fig. 2b-c). However, β-diversity of each groups’ gut microbiome analyzed by UniFrac principal coordinates analysis (PCoA) was significantly different among the groups (*p* = 0.001, 8 W versus (vs.) 1Y; *p* = 0.001, 8 W vs. 2Y; and *p* = 0.009, 1Y vs. 2Y; Permutational multivariate analysis of variance (PERMANOVA)) (Fig. 2d).

**Fig. 3:**
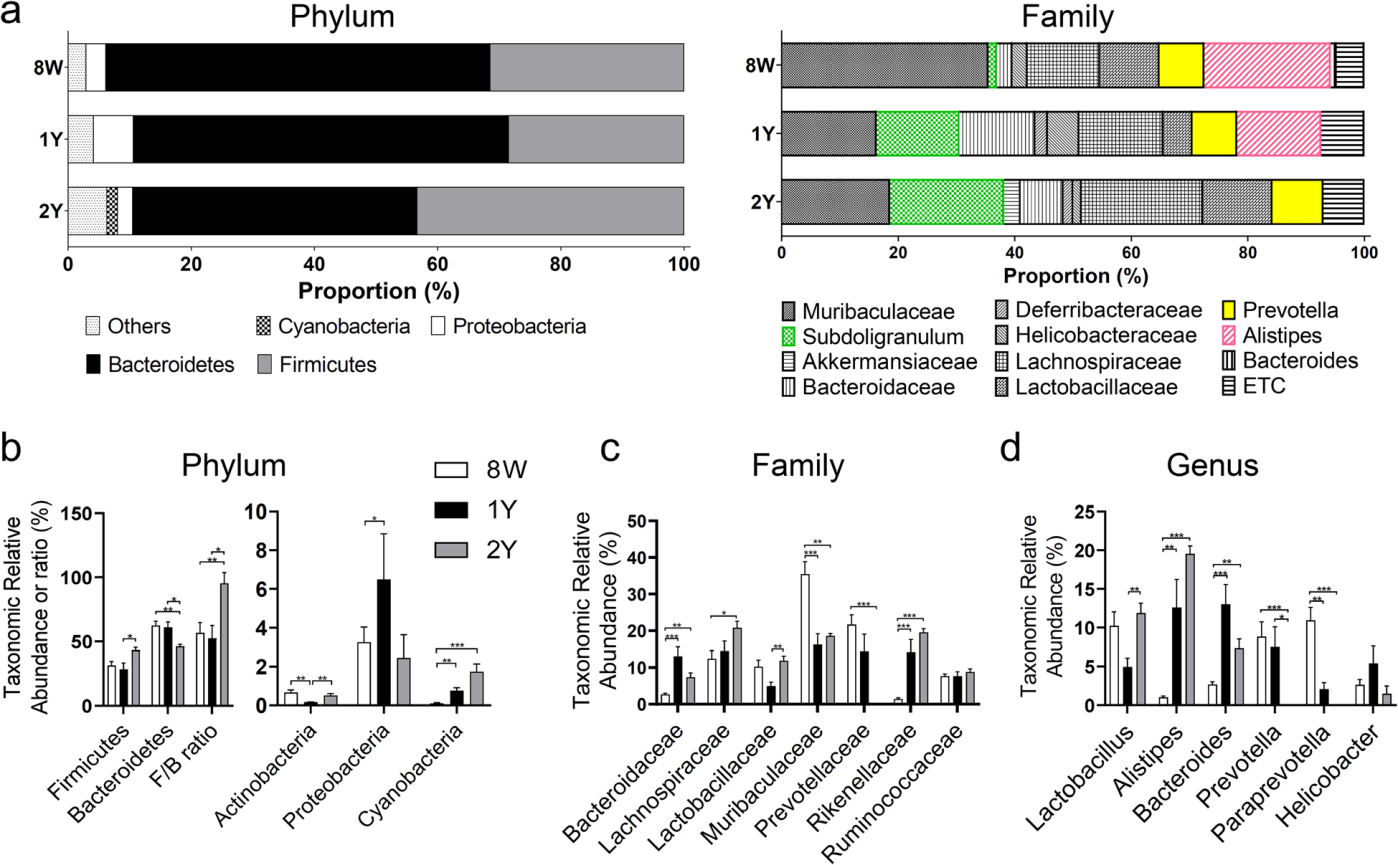
Taxonomic relative abundance according to phylum, family and genus. **a** Overall, taxonomic relative abundance in phylum and genus are shown for all groups. **b** In phylum, *Firmicutes* significantly increased (2Y versus (vs.) 1Y, *p* = 0.037) and *Bacteroidetes* reduced (2Y vs 8 W, *p* = 0.004; 2Y vs 1Y, *p* = 0.024) in the 2Y group compared to both 8 W and 1Y groups. *Proteobacteria* was increased (1Y vs. 8 W, *p* = 0.049) and *Actinobacteria* was decreased (1Y vs 8 W, *p* = 0.002; 1Y vs 2Y, *p* = 0.008) in the 1Y group. *Cyanobacteria* was increased in the aged group (1Y and 2Y) compared to the 8 W group (*p* = 0.002 and *p* < 0.001, respectively). **c** In family, *Bacteroidaceae* and *Rikenellaceae* were significantly more abundant and *Muribaculaceae* was significantly reduced in the aged group (1Y and 2Y) compared to the 8 W group (all *p <* 0.05). *Prevotellaceae* was significantly reduced in the 2Y group than in 8 W group (*p* < 0.001). **d** In genus, the aged group (1Y and 2Y) were increased *Alistipes* and *Bacteroides*, and decreased *Paraprevotella* compared to the 8 W group (all *p* < 0.01). The 2Y group revealed significantly decreased *Prevotella* than both 8 W and 1Y groups (all *p* < 0.05). *p < 0.05, **p < 0.01, and ***p < 0.001 by Kruskal–Wallis test followed by Dunn’s post hoc test. Data are presented as means ± standard error

Thereafter, we analyzed compositional differences of gut microbiome at the level of phylum, family, and genus of each group (Fig. 3a). At the phylum level (Fig. 3b), the 2Y group exhibited relatively more *Firmicutes* than the 1Y group (*p* = 0.037), and decreased *Bacteroidetes* compared to both 8 W and 1Y groups (*p* = 0.004 and *p* = 0.024, respectively; Kruskal–Wallis test followed by Dunn’s post hoc test). *Firmicutes*/*Bacteroidetes* (F/B) ratio was greater in the 2Y group than the 8 W and 1Y groups (*p* = 0.004 and *p* = 0.024, respectively; Kruskal–Wallis test followed by Dunn’s post hoc test). The 1Y group revealed relatively higher proportion of *Proteobacteria* (1Y vs. 8 W, *p* = 0.049), and lower proportion of *Actinobacteria* (1Y vs 8 W, *p* = 0.002; 1Y vs 2Y, *p* = 0.008; Kruskal–Wallis test followed by Dunn’s post hoc test). The aged group (1Y and 2Y) showed a significantly abundant *Cyanobacteria* compared to the 8 W group (*p* = 0.002 and *p* < 0.001, respectively). At the family level (Fig. 3c), the aged group had high proportion of *Bacteroidaceae* and *Rikenellaceae*, and low proportion of *Muribaculaceae* compared to the 8 W group (all *p* < 0.05). Family *Prevotellaceae* reduced with aging and the 2Y group showed a significant reduction compared to the 8 W group (*p* < 0.001). At the genus level (Fig. 3d), the aged group showed higher proportion of *Alistipes* and *Bacteroides,* and lower proportion of *Paraprevotella* than the 8 W group (all *p* < 0.05). The 2Y group revealed significant reduction in *Prevotella* compared to both 8 W and 1Y groups (all *p* < 0.05; Kruskal–Wallis test followed by Dunn’s post hoc test).

**Fig. 4:**
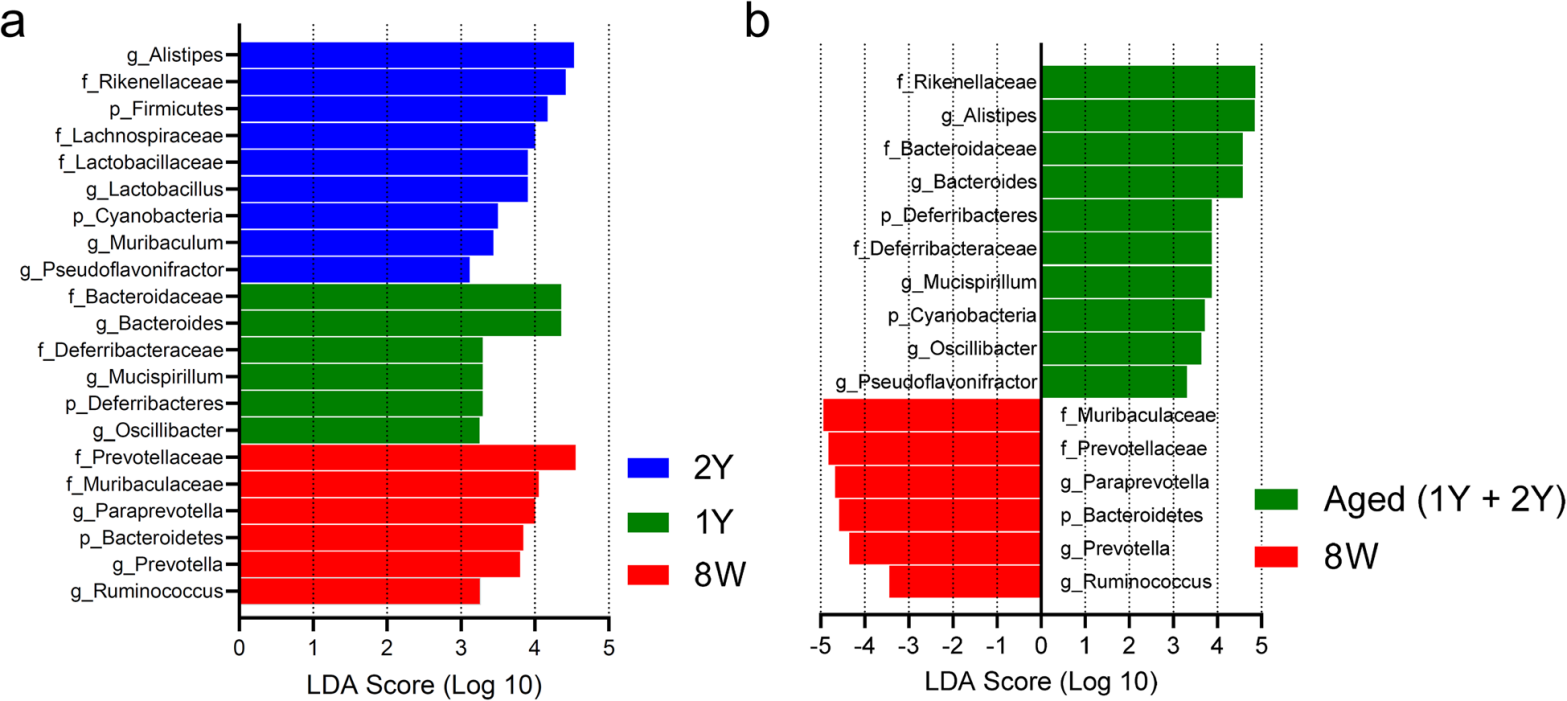
Linear discriminant analysis (LDA) effect size (LEfSe) performed on the microbial community relative abundance data. Kruskal-Wallis test produced *p* < 0.05 and an LDA score of > 3.0. **a** The 2Y group is shown as blue, 1Y as green, and 8 W as red. **b** Combined aged (1Y + 2Y) group is shown green, and 8 W young group as red

The linear discriminant analysis (LDA) of effect size (LEfSe) analysis was performed to explore the specific taxa that had significant difference among the 8 W, 1Y, and 2Y groups (Fig. 4a), or between the aged (1Y and 2Y) and 8 W young (Fig. 4b) groups. The relative abundance of nine microbial taxa, such as *Alistipes* (genus), *Firmicutes* (phylum), *Cyanobacteria* (phylum), in the 2Y group was higher compared to both 1Y and 8 W groups. Six microbial taxa, such as *Bacteroidetes* (phylum) and *Prevotella* (genus), were significantly enriched in 8 W group. For additional analysis, we combined the data from 1Y and 2Y groups into the aged group. Ten microbial taxa in the aged group, such as *Alistipes* (genus) and *Cyanobacteria* (phylum), were higher in abundance compared to the 8 W group, whereas six microbial taxa in the young 8 W group, such as *Paraprevotella* (genus), *Bacteroidetes* (phylum), and *Prevotella* (genus), were higher in abundance compared to the aged group.

**Fig. 5:**
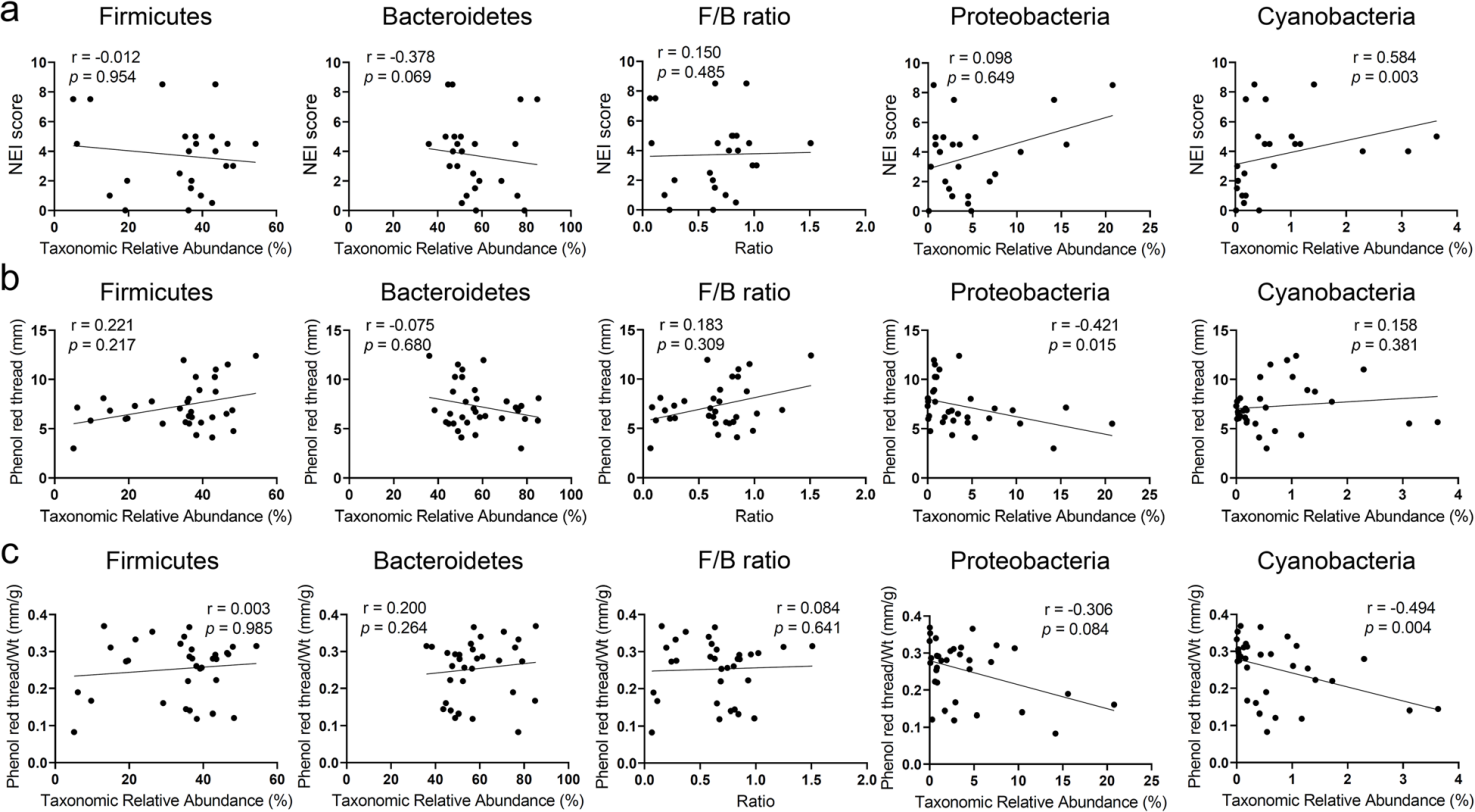
Spearman’s rank correlation analysis between dry eye indices and gut microbiome at the phylum level. **a** NEI score showed significant positive relation with *Cyanobacteria* (*r =* 0.584, *p* = 0.003). **b** Tear secretion was negatively related with *Proteobacteria* (*r =* − 0.421, *p* = 0.015). **c** Tear secretion with body weight adjustment showed negative relation with *Cyanobacteria* (*r =* − 0.494, *p* = 0.004)*.* NEI: National Eye Institute

**Fig. 6:**
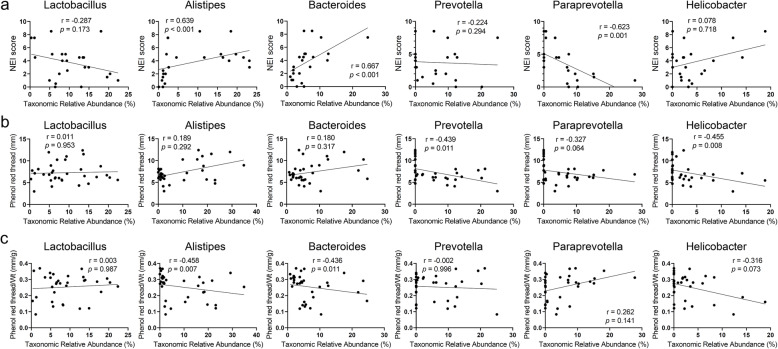
Spearman’s rank correlation analysis between dry eye indices and gut microbiome at the genus level. **a** NEI score showed significant positive relation with *Alistipes* (*r =* 0.639, *p* < 0.001) and *Bacteroides* (*r =* 0.669, *p* < 0.001), and negative relation with *Paraprevotella* (*r =* − 0.623, *p* = 0.001). **b** Tear secretion showed negative relation with both *Prevotella* (*r =* − 0.439, *p* = 0.011) and *Helicobacter* (*r =* − 0.455, *p* = 0.008). **c** Tear secretion with body weight adjustment showed negative relation with *Alistipes* (*r =* − 0.458, *p* = 0.007) and *Bacteroides* (*r =* − 0.436, *p* = 0.011). NEI: National Eye Institute

Next, we investigated the relationship between aging-dependent changes of gut microbiome and dry eye severity signs using Spearman’s rank correlation test. In phylum level (Fig. 5), corneal staining score was positively related with *Cyanobacteria* (*r =* 0.584, *p* = 0.003). Tear secretion had negative relation with *Proteobacteria* (*r =* − 0.421, *p* = 0.015). BW adjusted tear secretion was negatively correlated with *Cyanobacteria* (*r =* − 0.494, *p* = 0.004)*.* In genus level (Fig. 6), corneal staining score was positively related with *Alistipes* (*r =* 0.639, *p* < 0.001) and *Bacteroides* (*r =* 0.669, *p* < 0.001), and negatively related with *Paraprevotella* (*r =* − 0.623, *p* = 0.001). Tear secretion had negative relation with *Prevotella* (*r =* − 0.439, *p* = 0.011) and *Helicobacter* (*r =* − 0.455, *p* = 0.008). However, BW adjusted tear secretion was negatively correlated with *Alistipes* (*r =* − 0.458, *p* = 0.007) and *Bacteroides* (*r =* − 0.436, *p* = 0.011).

Finally, we performed partial rank correlation analysis to adjust the confounding age factor. In phylum level, only *Proteobacteria* significantly affected corneal staining score (*r =* 0.418, *p* = 0.047), tear secretion (*r =* − 0.423, *p* = 0.016), and tear secretion with BW adjustment (*r =* − 0.371, *p* = 0.037). In genus level, corneal staining score was positively related to *Helicobacter* (*r =* 0.423, *p* = 0.046). Both tear secretion with and without adjustment for BW were negatively related to *Prevotella* (*r =* − 0.444, *p* = 0.011; *r =* − 0.472, *p* = 0.006; respectively), *Paraprevotella* (*r =* − 0.367, *p* = 0.039; *r =* − 0.368, *p* = 0.038; respectively), and *Helicobacter* (*r =* − 0.455, *p* = 0.009; *r =* − 0.399, *p* = 0.024; respectively).

## Discussion

This study demonstrates that (1) aging-dependent gut dysbiosis is present in B6 male mouse model and (2) dry eye severity is correlated with the gut dysbiosis.

In dry eye disease and Sjögren’s syndrome patients, *Bacteroidetes*, *Actinobacteria*, and *Bifidobacterium* were correlated with dry eye severity signs. Sjögren’s syndrome patients showed significant gut dysbiosis compared to both healthy and environmental dry eye subjects [17]. In the autoimmune dry eye mouse model, mice fed with a specific probiotics formula resulted in reduced corneal erosion and increased tear volume. A decrease in the inflammatory cytokine, interleukin (IL)-1β, and increase in the anti-inflammatory cytokine, IL-10, were observed in the probiotics intake group [21]. These seem to be caused by the downregulation of antigen-presenting cells related to gut microbiome changes [21]. Kawashima et al. also reported that dietary supplementation with *E. faecium* WB2000 mixed with fish oil for 8 weeks improved objective and subjective symptoms with increased tear production in dry eye disease patients [22]. Taken together, dry eye severity signs are associated with gut microbiota dysbiosis, and the consumption of probiotics supplements can alleviate dry eye disease.

In this study, we observed more significant dry eye and related ocular surface damages in aged mice (1Y and 2Y) compared to the young group (8 W) in the B6 male mouse model. Corneal staining score of 1Y group was comparable to that of 2Y group. This is in line with previous studies that observed equivalent degree of corneal epithelial defects between 1 and 2 years of age [23], and similar corneal irregularities of 6 to 9 month-old B6 mice when compared to those of 2 year old B6 mice [24]. Aging-related inflammation increases with age, but not in a linear incremental manner [24], and the ocular surface change appears to reach a plateau between 1 and 2 years of age.

We found that the α-diversity of the gut microbiome did not change with age. However, β-diversity and microbiome composition were significantly different according to age, and this difference was clearly observed in the PCoA plot (Fig. 3a). In addition, the distribution in the PCoA plot was very different between 8 W and 2Y groups, whereas the 1Y group lied somewhere in between those groups.

Dysbiosis has been reported in several metabolic disorders such as obesity and hypertension, and appears to increase the relative abundance of F/B ratio [25–27]. High F/B ratio is associated with lower butyrate production in the gut [27, 28]. Butyrate, one of short-chain fatty acids (SCFAs), is a preferred energy source for intestinal epithelial cells and has several beneficial clinical effects such as, strengthening the immune system, reducing inflammation, and regulating metabolism [29]. In ocular diseases, high F/B ratio was observed in experimental autoimmune uveitis and age-related macular degeneration mouse models [30, 31]. In this study, *Bacteroidetes* was more dominant in the 8 W group, while *Firmicutes* was more prevalent in the 2Y group. In congruence with previous study [32], the F/B ratio increased with age where the 2Y group showed a significantly higher ratio than the 8 W and 1Y groups. These results suggest that gut dysbiosis increases with aging.

Phylum *Proteobacteria* is a known microbial signature for gut dysbiosis [25]. Gut dysbiosis facilitates anaerobic glycolysis of surface colonocytes, which induces increment of gut epithelial oxygenation, disrupts anaerobiosis in the lumen, and consequently promotes an expansion of facultative anaerobic *Proteobacteria* [33]. Immunomodulatory cytokine IL-10 deficient mice showed spontaneous development of colitis and had relatively higher proportion of *Proteobacteria* than wild-type mice [34]. Oral administration of *Helicobacter typhlonius* (phylum *Proteobacteria*) triggered tumor necrosis factor (TNF)-α and promoted colitis in Tbx21^−/−^Rag2^−/−^ ulcerative colitis mice [35]. Increased prevalence of *Proteobacteria* was observed in metabolic or immune disorders including type II diabetic mellitus, obesity, and systemic lupus erythematosus [36–38]. Among ocular diseases, *Proteobacteria* was increased in Behçet’s disease and age-related macular degeneration [31, 39]. In this study, the abundance of phylum *Proteobacteria* was increased in the 1Y group. Moreover, correlation analysis revealed that phylum *Proteobacteria* was positively related to corneal staining and negatively related to tear secretion after age adjustment. Genus *Helicobacter* also showed a negative correlation with tear secretion regardless of BW adjustment. This genus has been reported to be associated with enteritis and systemic infectious diseases [40].

Phylum *Cyanobacteria* is a bacteria presumably to be involved in the onset of neurodegenerative disease such as Parkinson disease. *Cyanobacteria* produces neurotoxin b-N-methylamino-L-alanine that may trigger neurodegeneration by promoting mitochondrial dysfunction, protein misfolding, and innate immune responses in genetically susceptible individuals [41]. This phylum was observed to relatively increase in aged mice, and the associated gut dysbiosis was also observed in two different mouse models of progeria where fecal microbiota transplantation from wild-type mice enhanced their lifespan and health [42]. BW adjusted tear secretion was negatively correlated with the *Cyanobacteria*. Both afferent and efferent neural functions modulating tear production and neurodegeneration induced by *Cyanobacteria* may play etiological roles [1].

Of note, genus *Paraprevotella* decreased with aging as reported previously in another study [43]. A decrease in this genus was associated with an increase in corneal staining score. Reduced genus *Paraprevotella* has been reported in patients with autism spectrum disorder compared to healthy subjects, in low-functioning older adults than in high-functioning older adults, and in sedentary women than in active women [44–46]. In addition, genus *Paraprevotella* showed negative association with disease staging and motor function of Parkinson disease [47]. This genus produces succinate and acetate as major fermentation products, and only two associated species have been discovered. As described above, the reduction of genus *Paraprevotella* is related to several diseases but the potential impact on human health is still unknown [46, 48], and it may be connected to the production of acetate, one of the SCFAs.

We also observed that the relative abundance of genus *Bacteroides*, regarded as an opportunistic pathogen, was significantly increased with age, in agreement with previous studies [49–51]. This genus has been shown to produce virulent factors in the form of polysaccharide that is involved in the destruction of tissues and formation of abscess [52]. The genus *Bacteroides* was positively correlated with corneal staining score, which is possibly associated with disruptive gut barrier and systemic inflammation.

A suggested pathophysiology of gut dysbiosis associated with dry eye disease may involve modulating effector T cells directly or via either dendritic cell activation or disturbing gut-derived metabolites [19]. In this study, we found that microbial taxa associated with dysbiosis change with age, and that these microbial taxa were associated with dry eye severity signs. With aging, phylum *Proteobacteria* and genus *Helicobacter* (a genus included in phylum *Proteobacteria*) were positively associated with corneal staining score and negatively associated with tear secretion after age adjustment. The modulation of phylum *Proteobacteria* may be relevant in the treatment of age-related dry eye disease.

Our study has several limitations. First, a small number of animals were used, and the corneal staining score was not measured in all mice. Therefore, the results may be insufficient to represent each group. However, this study can be used as a pilot study for future research. Second, this study was conducted with only male mice. The female sex is one of the potent risk factors for dry eye disease due to hormonal effects [53]. Therefore, it may not be possible to extend the results to female. Third, this was an animal study conducted with mice. However, B6 mice are frequently used in aging research because they develop all the hallmarks of dry eye clinical features including corneal barrier dysfunction, loss of goblet cells, meibomian gland dysfunction, increased dry eye related cytokine expressions, dry eye signs with age, and inflammatory cell infiltrations in lacrimal gland associated with aging [54–56]. Fourth, we did not co-house mice or use littermates. We included mice of different ages; therefore, we could not use the same littermates. Moreover, mice that have passed the weaning period, especially males, tend to fight until death when co-housed, and so, we could not co-house them [57]. For these reasons, a co-housing or littermate method could not be applied in previous studies comparing gut microbiome of young and old mice [58, 59]. Fifth, relative abundance may not be as accurate as absolute abundance [60]. The overall abundance of bacteria in the gut is likely to also change with age, thereby just showing relative abundance may overlook important key findings. Lastly, we evaluated the association between gut microbiome changes and dry eye severity signs, and we did not reveal any cause-and-effect relationship. Analysis of serum inflammatory cytokines, stool SCFA, or histological changes in the ocular surface with aging will be needed in future studies to strengthen the association of age-related microbiome changes to dry eye severity. Probiotics have been observed to improve dry eye disease severity, suggesting that regulation of gut microbiota might aid future discovery of effective treatment for dry eye disease [21, 22].

## Conclusions

Several microbial taxa change in aged mice are related to gut dysbiosis or chronic inflammation, which are possibly associated with the severity of dry eye. In aged mice, phylum *Proteobacteria*, *Cyanobacteria*, and genus *Bacteroides* increased while genus *Paraprevotella* decreased. These microbial taxa were associated with increased corneal staining score or decreased tear volume. Especially, phylum *Proteobacteria* was positively associated with corneal staining score. Modulation of gut dysbiosis may be a new therapeutic strategy for age-related dry eye disease.

## Method

The experimental protocol was approved on May 29, 2017 by the Institutional Animal Care and Use Committee of the Seoul National University Biomedical Research Institute (IACUC no. 19–0076-S1A0). Animal experiments were performed in accordance with the ARVO Statement for Use of Animals in Ophthalmic Vision and Research and ARRIVE guidelines. It is known that B6 mice have increased corneal erosion at 6–9 months of age, and histological changes in the lacrimal glands are evident at 12 months of age [24, 61]. Moreover, mice older than 24 months have a have a higher chance of natural death caused by aging [62]. Therefore, we set 12 and 24 months of age as the time points of the analysis and compared them with 8-week-old mice as controls.

### Animals

A total of 33 B6 male mice were used in this study. Fifteen 8 W, ten 1Y, and eight 2Y mice were included. All mice were purchased from the same company (KOATECH; Pyeongtaek, Gyeonggi-do, South Korea) at 6-weeks of age, and freely fed a normal diet of laboratory rodent chow (38,057; Cargill Agri Purina, Inc., Seongnam, South Korea). Mice cages were in a specific pathogen-free facility at Seoul National University Hospital Biomedical Research Institute (Seoul, Korea), maintained at 22–24 °C with 55 ± 5% relative humidity, and given food and water ad libitum. After the study, the mice continued to be observed without sacrificing for further study related to aging-related dry eye disease.

### Clinical evaluation

The corneal staining and tear secretion test were performed under anesthesia (using a mixture of xylazine and zoletil at a ratio of 3:1). The corneal staining was evaluated first, and the tear secretion was measured at least 24 h apart. The corneal staining scores were blindly assigned by a single experienced ophthalmologist (CH. Y.) according to the National Eye Institute (NEI) grading scale [63]. A drop of 0.25% fluorescein dye was applied to the conjunctival sac for 30 s, then the ocular surface was gently washed with 1 mL of normal saline, and corneal staining was evaluated using a microscope (Olympus SZ61; Olympus Corporation, Tokyo, Japan) under cobalt blue illumination. For the tear secretion test, phenol red-impregnated cotton threads (FCI Ophthalmics, Pembroke, MA, USA) were inserted into the lateral canthus of mice for 60 s. The amount of tear secretion was determined by measuring the length of the wet thread in millimeters. The correction value obtained by dividing the amount of tear secretion by the BW of the mouse was also calculated [64]. Corneal staining score was evaluated in ten 8 W, six 1Y, and eight 2Y mice, and tear secretion test was performed in all mice. The average values of corneal staining scores and tear secretion results from both eyes were used for the analyses.

### Fecal microbiota analysis

All collected feces were stored at − 80 °C until they were referred to Chunlab, Inc. (Seoul, Korea) for microbiota analysis as previously described [17]. Total DNA extraction was performed using the FastDNA® SPIN Kit for Soil (MP Biomedicals, USA), in accordance with the manufacturer’s instruction. Polymerase chain reaction (PCR) amplification was performed using extracted DNA and bacterial PCR primers 341F (5′-TCGTCGGCAGCGTC-AGATGTGTATAAGAGACAG-CCTACGGGNGGCWGCAG-3′; underlining sequence indicates the target region primer) and 805R (5′-GTCTCGTGGGCTCGG-AGATGTGTATAAGAGACAG-GACTACHVGGGTATCTAATCC-3′) targeting V3-V4 regions of 16S rRNA. The reaction conditions for the first PCR amplification were as follows: 3 min of initial denaturation at 95 °C, 25 cycles of 30 s’ denaturation at 95 °C, 30 s primer annealing at 55 °C, 30 s elongation at 72 °C, and final extension at 72 °C for 5 min. The second PCR amplification was performed using i5 forward primer (5′-AATGATACGGCGACCACCGAGATCTACAC-XXXXXXXX-TCGTCGGCAGCGTC-3′; X indicates the barcode region) and i7 reverse primer (5′-CAAGCAGAAGACGGCATACGAGAT-XXXXXXXX-GTCTCGTGGGCTCGG-3′) for attaching the Illumina NexTera barcode. The second amplification conditions were the same as those described for the first reaction except only eight amplification cycles were performed. Amplification was confirmed using gel electrophoresis on 1% agarose gel and visualized under a Gel Doc system (BioRad, Hercules, CA, USA). The product size and quality were assessed on a Bioanalyzer 2100 (Agilent, Palo Alto, CA, USA) using a DNA 7500 chip. Mixed amplicons were pooled and sequencing was performed using an Illumina MiSeq Sequencing system (Illumina, Inc., San Diego, CA, USA) according to the manufacturer’s instruction at Chunlab, Inc. (Seoul, Korea). The EzBioCloud database (http://ezbiocloud.net) was used for taxonomic classification after chimera check. To detect chimera on reads that contain lower than 97% best hit similarity rate, UCHIME and the non-chimeric 16S rRNA database from EzBioCloud were used [17]. The dataset was normalized to the lowest number of read counts (28903 reads per sample) for further analysis.

### Statistical analysis

α-diversity analysis expressed with the observed OTUs, Chao1, Shannon, and β-diversity analysis expressed with generalized UniFrac were carried out using EZBioCloud, a bioinformatics cloud platform of ChunLab Inc. (Seoul, Korea). PCoA was performed to visualize differences in the samples at genus and species level. PERMANOVA was used to evaluate significance in UniFrac PCoA. Compositional abundance differences from each sample were identified by analysis using LEfSe and the Kruskal-Wallis test [65]. Taxa with more than 1% abundance in at least one group were included in the analysis. Only those taxa that showed a *p*-value < 0.05 and log LDA score ≥ 3 were used as thresholds. For multiple comparisons, the Kruskal–Wallis test followed by Dunn’s post hoc test was used. Spearman’s rank correlation and partial rank correlations were used to analyze the relationship between measures. Spearman’s rank correlation analysis between dry eye indices and clinically important microbials with significant differences among groups was performed. Partial rank correlation analysis was performed to identify independent significant microbials affecting dry eye signs after adjustment of confounding age factor. Statistical analyses were performed using GraphPad Prism software (version 8.2.0; GraphPad Software, La Jolla, CA, USA) and SPSS Statistics 20.0 (IBM Corporation, NY, USA). Differences were considered statistically significant at *p* < 0.05.

## Supplementary Information

**Additional file 1: Table S1.** Corneal staining score, tear secretion, and body weight adjusted tear secretion of each mouse.

## Data Availability

The datasets used and/or analyzed during the current study are available from the corresponding author on reasonable request.

## Abbreviations

1Y: One-year old
2Y: Two-year-old
8 W: Eight-week-old
B6: C57BL/6
BW: Body weight
F/B: *Firmicutes*/*Bacteroidetes*
IL: Interleukin
LDA: Linear discriminant analysis
LEfSe: Linear discriminant analysis of effect size
NEI: National Eye Institute
OTU: Operational taxonomic unit
PCoA: Principal coordinates analysis
PCR: Polymerase chain reaction
PERMANOVA: Permutational multivariate analysis of variance
SCFA: Short-chain fatty acid
SD: Standard deviation
Th1: T helper 1 cell
Th17: T helper 17 cell
TNF: Tumor necrosis factor
T_reg_: Regulatory T cell
vs.: Versus
