# Supplementary material for: Association between aging-dependent gut microbiome dysbiosis and dry eye severity in C57BL/6 male mouse model: a pilot study
Source: BMC Microbiol. 2021 Apr 9;21:106. doi: 10.1186/s12866-021-02173-7 (PMC8033717; doi:10.1186/s12866-021-02173-7)
Supplement: Supplementary file 1 — Additional file 1: Table S1. Corneal staining score, tear secretion, and body weight adjusted tear secretion of each mouse. [file 12866_2021_2173_MOESM1_ESM.docx]

**Supplementary table 1. Corneal staining score, tear secretion, and body weight adjusted tear secretion of each mouse.**

| Group | Number | NEI score | Tear secretion (mm) | Tear secretion /BW (mm/g) |
| --- | --- | --- | --- | --- |
| 8W | 1 | 0 | 8.035 | 0.366 |
|  | 2 | 1.5 | 6.707 | 0.305 |
|  | 3 | 2.5 | 7.046 | 0.321 |
|  | 4 | 1 | 5.625 | 0.256 |
|  | 5 | 1 | 6.826 | 0.311 |
|  | 6 | 0 | 5.999 | 0.273 |
|  | 7 | 3 | 6.500 | 0.296 |
|  | 8 | 0.5 | 6.160 | 0.281 |
|  | 9 | 2 | 6.049 | 0.275 |
|  | 10 | 2 | 6.165 | 0.281 |
|  | 11 | NA | 6.873 | 0.313 |
|  | 12 | NA | 8.098 | 0.369 |
|  | 13 | NA | 6.281 | 0.286 |
|  | 14 | NA | 7.763 | 0.354 |
|  | 15 | NA | 7.304 | 0.333 |
| 1Y | 1 | 8.5 | 5.506 | 0.160 |
|  | 2 | 4.5 | 7.147 | 0.189 |
|  | 3 | 7.5 | 5.813 | 0.166 |
|  | 4 | 4.5 | 4.341 | 0.119 |
|  | 5 | 7.5 | 2.995 | 0.083 |
|  | 6 | 5 | 4.106 | 0.132 |
|  | 7 | NA | 10.238 | 0.291 |
|  | 8 | NA | 7.728 | 0.220 |
|  | 9 | NA | 11.959 | 0.340 |
|  | 10 | NA | 8.918 | 0.254 |
| 2Y | 1 | 5 | 10.258 | 0.261 |
|  | 2 | 5 | 5.661 | 0.144 |
|  | 3 | 4.5 | 11.521 | 0.293 |
|  | 4 | 8.5 | 8.755 | 0.222 |
|  | 5 | 3 | 4.761 | 0.121 |
|  | 6 | 4 | 11.004 | 0.279 |
|  | 7 | 4.5 | 12.393 | 0.315 |
|  | 8 | 4 | 5.517 | 0.140 |

NEI: National Eye Institute; BW: body weight; W: week; Y: year; NA: not available
